# Inflammatory Bowel Disease and Thrombosis

**DOI:** 10.5505/tjh.2012.04557

**Published:** 2012-06-05

**Authors:** Ahmet Tezel, Muzaffer Demir

**Affiliations:** 1 Trakya University, School of Medicine, Department of Gastroenterology, Edirne, Turkey; 2 Trakya University, School of Medicine, Department of Hematology, Edirne, Turkey

**Keywords:** Inflammatory bowel disease, Thrombosis

## Abstract

Inflammatory Bowel Disease (IBD) is a group of chronic and relapsing inflammatory disorders of the gastrointestinalsystem. In these cases, findings are detected in extraintestinal systems also. There is a tendency for thrombotic eventsin IBD, as in the other inflammatory processes. The pathogenesis of this thrombotic tendency is multidimensional,including lack of natural anticoagulants, prothrombotic media induced via the inflammatory process, long-termsedentary life style, steroid use, surgery, and catheter placement. The aim of this review was to highlight the positiverelationship between IBD and thrombotic events, and the proper treatment of at-risk patients.

## Abstract (tr)

İnflamatuvar Bağırsak Hastalıkları gastrointestinal sistemin kronik, tekrarlayıcı inflamatuvar hastalıklarıdır. Bu olgularda GİS yanında diğer sistemleri de ilgilendiren bulgular saptanır. Diğer inflamatuvar patolojilerde olduğu gibi inflamatuvar Bağırsak hastalıklarında da trombotik olaylara eğilim artmaktadır. Tromboza yatkınlığın patogenezi çok boyutludur. Doğal antikoagülanlarda eksiklik, inflamatuvar sürecin yarattığı protrombotik ortam, uzun süreli hareketsizlik, steroid kullanımı, cerrahi girişim, kateter takılması tromboz gelişiminde suçlanmıştır. Bu derlemenin amacı İBH ve trombotik olaylar arasındaki ilişkiyi klinisyenlere hatırlatmak, tromboz gelişme riski olan olgularda gerekli önlemlerin alınmasını sağlamaktır.

## INTRODUCTION

Inflammatory bowel disease (IBD) is a group of disorders characterized by chronic, relapsing inflammation of the gastrointestinal system triggered by environmental factors in genetically susceptible individuals. The primary diseases that constitute IBD are ulcerative colitis (UC) and Crohn’s disease (CD). Systemic findings related to and not related to inflammatory activity may be observed in addition to gastrointestinal findings in both diseases. The etiopatho genesis of IBD is not been fully known. The genetics of IBD remain the greatest mystery. Clinical findings varyaccording to periods of inflammatory activity and remission.Drugs used for treatment cause additional clinicalfindings and morbidity.

The strong relationship between inflammation and hemostasis affects the progression and severity of disease in many inflammatory pathologies, including IBD, atherosclerosis,and rheumatoid arthritis [1]. The tendency for thrombosis in IBD has been know since 1936. Bargen and Barker [2] reported that life-threatening thromboembolic events develop in UC cases and that the tendencyfor thrombosis might be associated with thrombocyte dysfunctionor increased thromboplastin synthesis. Thereafter,other case reports and prospective studies reported thepotential for thrombosis to develop in venous and arterialsystems [3,4,5,6,7].

The present review aimed to evaluate the relationshipbet ween IBD and thrombosis. Inconsistent results, variation in study design, and insufficient clarification of the development of thrombosis in IBD complicate the issue.

Answering following questions may help under standing the relationship between IBD and thrombosis better.

1. Is there a real tendency to thrombosis in IBD? What is the magnitude of the problem?

2. With which mechanism does thrombosis develop in IBD?

3. Is there a relationship between thrombosis development and disease activity?

4. Can thrombotic pathologies be prevented?

5. Can anticoagulant agents be used in treatment of the disease?

**Thrombosis tendency in IBD**

Thrombosis tendency is accepted to increase in IBD.Thrombosis is encountered in arterial and more commonly in venous system besides micro vascular mucos al thrombosis.Thrombosis arises in deep crur al veins, pulmonary circulation,less commonly in cerebro vascular system, portalve in and retinal veins [3,4,5,6,7]. When we analyzed the magnitudeof the problem; frequency of systemic thromboembolism in IBD varies between 1-7% and reaches to 39%in some autopsy series [3,8,9]. In the study of Bernsteinet al. [10] deep venous thrombosis and pulmonary embolism are three fold higher among IBD cases compared to normal population. In addition to this, detection of the fact that IBD was less frequent among patients who have hemophilia and von Willebrand disease [11,12]. This prothrombiccondition was suggested to be able to play a role in pathogenesis of especially CD; multifoc al microscopiculcerations were detected even in healthy mucosa biopsiesmacroscopically [13].

Many factors that may lead to thrombosis are available in inflammatory bowel diseases and these factors are summarizedin Table 1 [14].

**Hereditary risk factors**

Factor V Leiden mutation, G20210A prothrombin gene mutation, C677T methylenet et rahydrofolate reductase gene mutation, Factor XIII gene mutation and plasminogenactivator inhibitor type 1 gene polymorphisms were hold to account in IBD and thrombophilia relationship[15]. Factor V Leiden mutation is a member of protein C natural anticoagulant system and is activated by bindingthromb in to thrombomodul in on the endothelial surface(APC). APC inhibits uncontrolled thrombosis formation by inactivating factor V and VIII. Arg506 Gln point mutation on factor V gene (factor V Leiden) makes factor Vresistant against inactivation by APC [14,16]. While frequency of factor V Leiden (FVL) is 2%-5% among Europeans,it was not found in native Africans, Native Americans and in Asia [17,18,19,20]. Its frequency was found as 7,9% as the results of studies carried out with healthy populations over the country [21]. FVL is the most common cause of thrombophilia and accounts for approximately 11-20% of acute venous thrombotic events [17].

IBD-FVL relationship was studied widely due to high frequency of FVL and because IBD is a prothrombotic condition.In a meta-analysis, a significant difference was not found between IBD cases and normal subjects in terms of FVL frequency [14]. However distinct results were reported in two studies. While FVL was found more frequentamong CD cases compared to UC cases and healthycontrols in the first one, FVL was found more frequentamong UC cases compared to CD cases and healthy controls in the study of Halsam et al. [22]. These different results were tried to be explained with low number of patients and difference in geographic distribution of themutation in meta-analysis of Papa et al. [14].

When thrombotic and non-thrombotic IBD cases were compared; Liebman et al. [19] found FVL in 36% of cases with thrombosis in their retrospective study. In the same study, they calculated IBD and FVL coincidence 23 fold greater compared to healthy subjects, risk of venous thrombosis development risk as 5-fold greater than nonthrombotic IBD cases. They suggested that FVL mutationshad significant contribution on venous thrombosis development in IBD cases. The fact that venous thromboembolismrisk increased in presence of FVL and especially before 45 years of age was confirmed in another study[23].

In a study reported from Turkey this year, it was stated that protein S and antithromb in III levels decreased along with protein C in active periods of both UC and CD, howe verno difference was reported in remission period. Additionally,activated protein C resistance was found indifferent from control group in active period and remission period. In this study, natural anticoagulant deficiency was emphasized to be able to lead to thrombophilia especially in cases in active period [24].

**Prothrombin G20210A gene mutation**

Prothrombin (PT) is a protein synthesized from the liver with the effect of vitamin K and converted to thrombin by prothrombinase complex in the course of coagulation.PT gene mutation develops by guanine adenine exchange at the 20210 position. PT level was found approximately 30% more in heterozygous subjects compared to healthy controls [14]. However a correlation was not found between IBD cases and PT gene mutation [9,25].

**C677T methylenetetrahydrofolate reductase gene mutation and hyperhomocysteinemia**

Hyperhomocysteinemia is a risk factor for both arterial and venous thromboembolism. Hyperhomocysteinemiamay arise as the result of a genetic defect (C677Tmethylenetetrahydrofolate reductase gene mutation) and also as the result of deficiency of cofactors like vitaminB12, B6 and folate [26]. Endothelial damage is in question following endotheli al dysfunction and thrombocyte activationin development of hyperhomocysteinemia - relatedthrombosis. Homocysteine converts to homocysteine, homocysteine thiolactone and mixed disulfides via rapidauto-oxidation in plasma, strong reactives like superoxideand hydrogen peroxide and this is more prominent especiallyin cases with hyperhomocysteinemia.

Homocysteine thiolactone and LDL conjugate and formaggregates, this leads to foam cell formation and contributes to reactive oxygen radical development. Reactive oxygenradicals lead to endothelial damage and dysfunction,this leads to excessive platelet activation and aggregation.Thrombosis in hyperhomocysteinemia was detected to berich of platelets. Reactive oxygen radicals also contributeto thrombosis development by oxidizing LDL, leading tolipid peroxidation and causing proliferation in vascularsmooth muscle cells [26].

Relationship between hyperhomocysteinemia andthrombosis development in IBD was researched widely.Hyperhomocysteinemia may be genetic in origin and also develops related to deficiency of cofactors like B6,folate and B12 vitamins that are effective on homocysteinemetabolism. Nutritional insufficiency, impairment inabsorption and decrease in their levels due to effects ofdrugs like sulfasalazine may be seen in IBD [27,28]. Intwo studies investigating IBD and hyperhomocysteinemiacoexistence, homocysteine levels were found to be significantlyhigher in IBD cases compared to healthy subjectshowever a relationship could not be found between hyperhomocysteinemiaand thrombosis development in bothstudies [29,30].

On the other hand, C677T methylenetetrahydrofolatereductase gene mutation was also investigated as the causeof hyperhomocysteinemia in IBD and conflicting resultswere reported. While C677T mutation was found significantlyhigher in IBD cases compared to healthy individualsin two studies held in Northern Europe [9,31], thereare studies reporting C677T mutation frequency in IBD ashigh as healthy individuals [28,32,33].

When evaluating C677T homozygosity and disease relationship, it should be kept in mind that this mutation may differ significantly according to ethnic and geographic factors. For example, while it is never seen in Africans, it was found highest among Italians and Spanish people [18-20%), moderate in northern countries like Germany, Holland,Norway, Ireland 87-10%) [34]. Thus it is obvious that C677T mutation in healthy individuals and subject swith IBD would give different results in the studies held inaforementioned countries or groups.

In general, absence of a correlation between FVL, prothrombingene mutation, C677T methylenetetrahydrofolatereductase gene mutations and IBD is not surprising.Although complex genetics of IBD has not been fully explained, association of genomic regions on chromosomes12 and 16 with diseases was shown [35]. On the other hand, prothrombic mutations were shown to be ongenes located on chromosomes 1 and 11 in the study of Vecchi et al. [32]. As the result, congenital thrombophilic disorders and IBD do not take place on the same genetic basis.

**IBD and Hemostasis Relationship**

Many factors related to the nature of the disease, metabolicoutcomes and drugs might lead to different conditionsin the course of IBD; they also may contribute tothrombosis formation [36].

**Inflammation and inflammatory activity **

Chronic intestinal inflammation triggered by luminal antigens in genetically susceptible subjects may be stated as the environmental factor in IBD. We can consider different wall structures of gram negative and anaerobic bacteria, bacteria products and dietary antigens as luminal antigens. Developing immunologic events, tissue damage and repair develop in a similar manner independently from the initiating factor in intestinal inflammation. Immune, mesenchymal and epithelial cells activated in intestinal wall implement tissue damage and repair via some soluble mediators. These mediators include cytokines, arachidonic acid metabolites and growth factors. While intestinal inflammation may be controlled in healthy individuals, down regulation of inflammation is not probable in IBD cases. If antigenic stimulus continues, inflammatory response arises gradually getting strong. This property (misfortune) is responsible for chronicity and disease in IBD cases. 

Many soluble mediators play an important role in pathogenesis of the disease in IBD. These may include arachidonic acid metabolites (prostagland in E2 [PGE2], thromboxane A2 [TXA2], and leukotriene B4 [LTB4]), platelet activating factor (PAF), cytokines (IL^-1^, IL^-2^, TNF-a, IL-8, IFN-g), and intercellular adhesion molecules (ICEM-I, etc.). On the other hand, cytokines like IL-4, IL-10 and IL-13 play roles in stabilization of inflammation, growth factors like transforming growth factor - beta (TGF-b) play roles in tissue repair [36]. PAF is synthesized and released by monocytes, neutrophils, basophils, mast cells, vascular endothelial cells and epithelial cells during inflammation [36]. Biologic effects of PAF are stimulation of thrombocyte and neutrophil aggregation, chemotaxis, stimulation of eicosanoid and IL-8 synthesis, contraction of smooth muscle cells and increasing mucosal chloride secretion. In active IBD cases, increased PAF was found in ileal and colonic mucosa biopsies and in feces [37]. Chiarantini et al. [12] detected an increase in aggregation of platelets. Although statistically insignificant, hyperaggregability was reported significantly in CD and no relationship was found between disease activity. Authors suggest that this may be related to PAF rather than TXA2 [11]. 

Systemic inflammation is accepted as a strong prothrombic stimulus. While inflammatory events increase procoagulant factors, they decrease natural anticoagulants and fibrinolytic activity [38]. Effects of inflammation on coagulation are summarized in Table 2. 

Hemostatic changes in the course of IBD are related to activity and severity of the disease. Alterations in hemostasis were widely investigated in experimental and clinical studies. One of the mainly affected compounds is platelets and changes both in activities and numbers of the platelets are detected. 

Kapsoritakis et al. [39] researched thrombopoietin (TPO) level that is a physiologic regulator of thrombopoiesis in IBD. While platelet count increases in active casesand this is used as an important indicator of inflammationnormalizing was detected in inactive cases, on the other hand, TPO level was found high independently from activity. A significant relationship could not be found between TPO level, and the platelet count, erythrocyte sedimentation rate, or C-reactive protein level. Authors reported that TPO could be related to procoagulant property of IBD besides platelet production [39]. Thrombocytosis and low volume platelets were proven to be associated with disease activity. Expression of activation proteins like P-selectin, GP53 and CD40-ligand increased both in UC and CD. Plasma CD40 ligand level was associated with prevalence of anatomic involvement. Presence of platelet aggregates was shown in mucosal biopsies of UC patients. Activation of platelets can be indicated with presence of platelet-leucocyte complexes in circulation [40].

Various steps of coagulation-fibrinolytic system were analyzed in active and remission periods of IBD. In the studies, it was reported that measurement of coagulation factor level was not healthy enough because IL 6 and TNF alpha and hepatic synthesis of these proteins could increase as acute phase reactants [8,41]. Thus prothrombin fragment 1+2 (F1+2) was reported to give better outcomes as the indicator of coagulation activation, fibrin degradation products (FDP) were reported to give better outcomes as the indicator of fibrinolytic system [39]. 

F1+2 and FDP levels were found high as indicating activation of coagulation and fibrinolytic system independently from inflammatory activity in IBD [8, 12,41]. FDP were found significantly low in cases using corticosteroids. Authors reported that corticosteroid usage caused risk for thrombosis development by decreasing fibrinolytic activity [41]. 

Another indicator of coagulation activation in IBD patients is thrombin increase. In other words, prothrombic property of the disease was proven by endogenous thrombin formation-another indicator of coagulation activation in IBD patients. That thrombin formation increased with CRP elevation or disease activity was shown with endogenous thrombin potential by Saibeni et al. Why thrombosis formation is more in active period is explained with these data [42]. 

As in all inflammatory events, fibrinolytic system inhibition is seen also in IBD patients. Decrease in tissue plasminogen activator (tPA)-fibrinolytic system activator- and increase in plasminogen activator inhibitor (PAI) -fibrinolytic system inhibitor- were detected [43]. Besides, frequencies of autoantibodies against compounds of coagulation system, especially phospholipids and protein S have also increased. However clinical importance of these antibodies is controversial. 

**Acquired risk factors **

Factors like longstanding immobilization, surgical intervention, corticosteroid treatment, and central venous catheter insertion considered among acquired factors are effective for thrombosis development not only in IBD but also in many other diseases. In a study carried out for detection of thrombosis risk, only corticosteroid use was found as a factor increasing thrombosis risk [9], on the other hand, in another study active illness, intestinal stenosis and fistula were reported as risk factors [23]. Age of thrombosis development was found earlier in both studies compared to non-IBD causes. Not only hospitalized patients but also patients followed up in outpatient setting in active period were shown to be risky for thromboembolism.Grainge et al. [44] investigated venous thromboembolism risk between periods of inflammatory bowel disease and found that cases in active period were more risky in pre-hospitalization period compared to the patients who were hospitalized. While this risk is prominent both in episodes and chronic active cases, it is less in cases in remission. [Hazard ratio 15.8 (9.8-25.5) during episodes in outpatient setting, 9.9 (6.7-14.7) in chronic active cases, 2.2 (1.5-3.2) in cases in remission, 3.2 (1.7- 2.3) during episodes in hospitalized cases, 2.8 (1.5-5.2) in chronic active cases, 1.7(1.1-2.9) in remission, 95% CI]. Thrombosis risk was shown to increase among IBD cases in subjects with acute medical diseases [45,46,47]. Using data of these multi-center randomized studies, IBD was also added to risk assessment scales used for detection of thrombosis risk in patients hospitalized due to acute medical diseases [48].

**May thrombosis development be prevented in IBD?**

Although IBD is accepted as a prothrombic clinical condition, a treatment preventing thrombosis development is not available in daily practice due to the fact that thrombosis physiopathology could not be fully enlightened, IBD-specific risk factors could not be fully determined except for corticosteroid use. Patient education is of great importance as general measures. Prevention of longstanding immobilization, counteracting smoking, and careful monitorization of subjects who underwent surgical intervention and who were inserted catheters are recommended. Besides, pharmacologic and mechanical thromboprophylaxis should definitely be made (with low molecular weight heparin) if there is not a contraindication in patients older than 40 years who are hospitalized and stay immobilized longer than 3 days. 

Heparin use in IBD 

Traditional treatment modalities like corticosteroids, 5-ASA/sulphasalazine, immunosuppressive drugs lead to additional morbidities in IBD and increase number of surgical interventions. In recent years, heparin use has taken place among alternative medical treatment methods with immunomodulatory drugs. 

Accepting IBD as a prothrombic condition, indicating intestinal microthrombosis even in histologically normal mucosa arised the opinion to benefit from anticoagulant effect of heparin [49]. Anticoagulant effect of heparin is inhibition of factor X in presence of antithrombin in plasma and inhibiting thrombin formation [48]. However heparin has immunomodulatory and anti-inflammatory effects besides well-known anticoagulant effect. These effects are summarized in Table 3. [49,50,51] 

Heparin is currently used as an alternative treatment in UC cases resistant to steroid. Three studies are available about this topic; heparin was used with an initial dose of 25.000-40.000 IV daily for 4-6 weeks, aPTT so as to be 60’’, 54%-90% remission was reported [52,53,54]. Some of the patients were administered subcutaneous heparin during 6 weeks-4 months and no relapses were reported after the drug had been discontinued. Heparin was tolerated well in these studies; the most frightening adverse reaction colonic bleeding was seen in 5% cases. 

Törkvist et al. [55] used dalteparin sodium (low molecular weight heparin) 5000 U SC bid during 12 weeks in 12 active UC cases resistant to steroid. They obtained clinical improvement in 11 (92%) cases and complete remission in 6 (50%) cases and reported no adverse effects. Authors suggested that low molecular weight heparin could be a good alternative due to its safety. 

On the other hand, in the prospective, randomized, double blind, placebo controlled, multicenter study of Bloom et al. [56] including 100 UC cases with mild or moderate activity, they reported that tinzaparin was not superior to placebo. In the meta-analysis of Shen et al. [57], both low molecular weight and standard heparin were reported not to be superior to conventional treatment and provided no additional benefits in ulcerative colitis cases. In addition to this, while importance of biologic agents in IBD treatment is gradually increasing in recent years, use of heparin and heparin derivatives for anti-inflammatorypurpose has played second fiddle [58].

**Conclusion**

The best example for interaction between hemostasisand inflammation is IBD. According to obtained experimentaland clinical data, it is accepted as a medical conditionthat thrombosis frequency increased. Thus the factthat thromboprophylaxis is necessary especially for subjectswho are planned to be hospitalized has been provenwith clinical studies. Thromboprophylaxis may be donewith pharmacologic and/or mechanical methods howeverpharmacologic methods are known to be superior.Although the most frightening adverse effect is bleeding,frequency of bleeding has not been increased comparedto placebo in cases that standard or low molecular weightheparin had been used. Besides, wound healing occursfaster due to anti-inflammatory and immunomodulatoryproperties of heparin molecules except for anticoagulantproperties and the disease remits easily. Evaluating use of anticoagulants in IBD from this point of view wouldbe beneficial. Heparins’, especially low molecular weightheparins’ introduction as an alternative treatment amongfuture treatment modalities is probable.

## CONFLICT OF INTEREST STATEMENT

The authors of this paper have no conflicts of interest, including specific financial interests, relationships, and/ or affiliations relevant to the subject matter or materials included.

## Figures and Tables

**Table 1:**
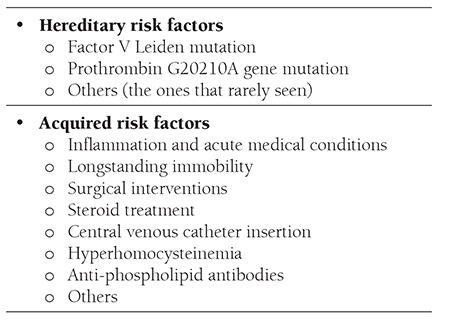
Hereditary and acquired prothrombic risk factors inIBD [14].

**Table 2:**
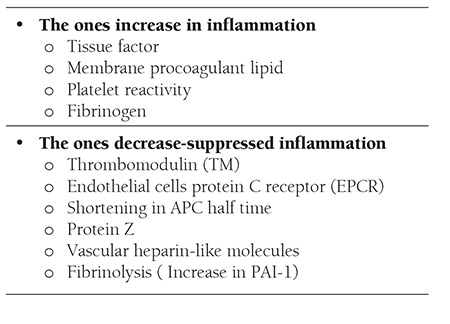
Effects of inflammation on hemostasis [38].

**Table 3:**
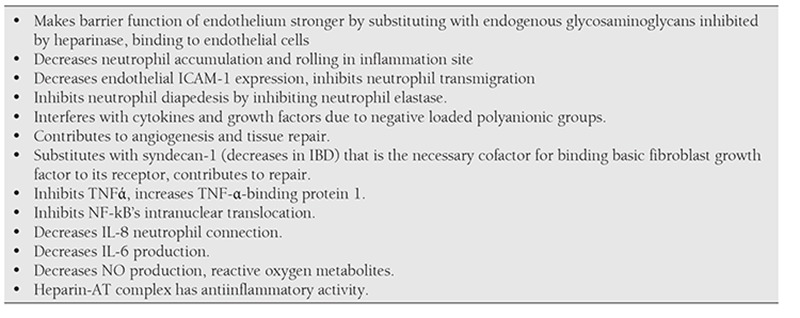
Immunomodulatory and anti-inflammatory effects of heparin [49-51].
